# Exploring the potential role of oxidative stress‐related genes in colorectal cancer recurrence and establishing a recurrence assessment system based on single‐cell and bulk RNA‐seq analysis

**DOI:** 10.1002/ctm2.1577

**Published:** 2024-02-08

**Authors:** Xin Gao, Jin Li, Qing‐Yu Wang, Zi‐Hui Wang, Si‐Jia Li, Lv‐Tao Zeng, Hong‐Lei Liu, Jian‐Ping Cai

**Affiliations:** ^1^ The Key Laboratory of Geriatrics, Beijing Institute of Geriatrics, Beijing Hospital, National Center of Gerontology, National Health Commission, Institute of Geriatric Medicine, Chinese Academy of Medical Sciences Beijing China; ^2^ Graduate School of Peking Union Medical College and Chinese Academy of Medical Sciences Beijing China; ^3^ School of Biomedical Engineering, Capital Medical University Beijing China; ^4^ Beijing Key Laboratory of Fundamental Research on Biomechanics in Clinical Application, Capital Medical University Beijing China

To the Editor:

Colorectal cancer (CRC) heterogeneity contributes to recurrence.^1^ Previous studies have reported a significant increase in reactive oxygen species (ROS) in CRC.^2^ ROS perform dual functions in CRC, being essential for cell survival at low levels, but cytotoxic at high levels and capable of influencing the tumour microenvironment (TME) heterogeneity, which contributes to CRC recurrence.^3^, ^4^ The role of oxidative stress and related genes in CRC TME heterogeneity and recurrence is not fully understood.

To address this issue, we analysed the scRNA‐seq dataset GSE144735 on normal tissue, tumour border tissue and tumour core tissue from six CRC patients (Figure S1A,B). The top five expressed genes in each cluster are shown in Figure S1C. The cells were classified into 20 clusters (Figure 1A). The differentially expressed genes of each cluster are presented in Figure 1B. The cell type annotation results are shown in Figure 1C. We found significant differences in the composition of certain cell types between the core and border of the tumour (false discovery rate (FDR) < .05; Figure 1D,E). The receptor communication strength among different cells in the tumour core was higher than that in the tumour border (Figure 1F,G). These findings indicated the existence of TME heterogeneity.

**FIGURE 1:**
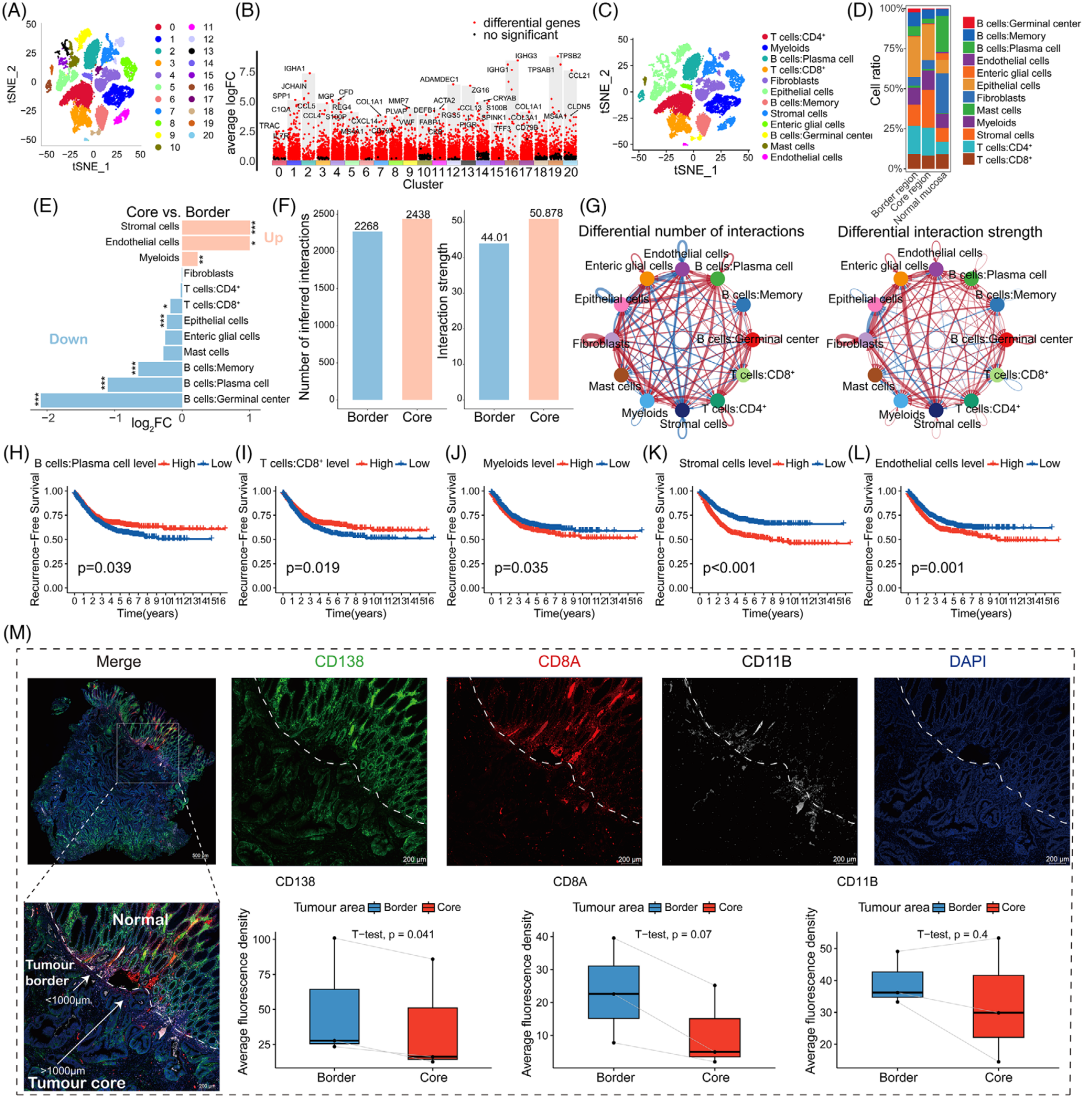
Analysis of microenvironmental heterogeneity in colorectal cancer. (A) t‐SNE plot based on scRNA‐seq data of colorectal cancer samples. The cells from all samples were grouped into 20 clusters. (B) Identification of differentially expressed genes for each cell cluster. The annotated genes represent the top two differentially expressed genes for each cluster. (C) Cell type annotation results for the 20 clusters. (D) Composition of immune cells in normal tissue, tumour core and tumour border. (E) Analysis of differences in cell abundance between the tumour core and border, in which FDR < .05 (*), FDR < .01 (**) and FDR < .001 (***). (F and G) Comparative analysis of the ‘Number of inferred interactions’ and ‘Interaction strength’ between the tumour core and border cells (the thickness of the line indicates the degree of change, with red indicating an increase and blue indicating a decrease). (H–L) Prognostic analysis of cell infiltration levels in 1320 patients with RFS (only cell types with *p* < .05 differences in RFS are displayed). (M) Representative micrographs of tumour border and core, as well as the expression differences of CD138 (plasma cells), CD8A (CD8^+^ T cells) and CD11B (myeloids) markers between tumour border and core.

We identified the top 10 expressed genes in cell types with significant abundance differences between the core and border. Using ssGSEA on bulk RNA‐seq data from 1320 CRC patients, which was integrated from TCGA_CRC, GSE17536 and GSE39582 datasets, we found that the abundance of five cell types significantly correlated with recurrence‐free survival (RFS) (*p* < .05; Figure 1H–L). These five cell types were identified as ‘heterogeneous cell types’. Furthermore, we validated the abundance of three immune cell types—CD138 (plasma cells), CD8A (CD8^+^ T cells) and CD11B (myeloid cells)—in three samples containing simultaneous normal, tumour border and tumour core tissues using mIHC. With the exception of CD11B, the results were consistent with the scRNA‐seq analysis (Figure 1M). We also collected 52 CRC samples and validated the relationship between the infiltration levels of these three immune cell types and patient recurrence in these cases (Tables S1 and S2). These immune cell infiltration levels were significantly associated with tumour grade and recurrence (Figure S2A–J).

**FIGURE 2:**
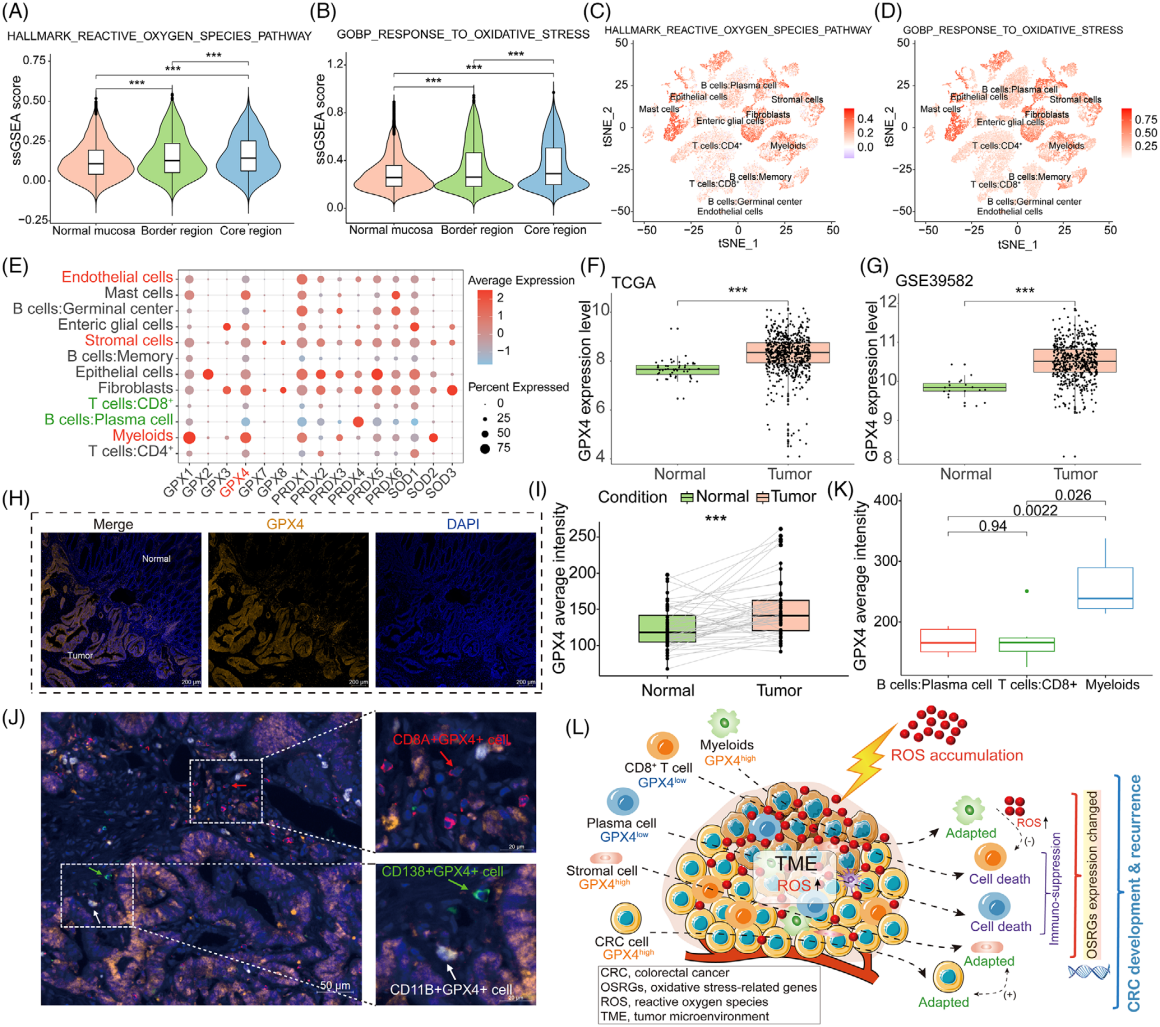
The role of oxidative stress in the heterogeneity of the colorectal cancer microenvironment. (A–D) Scoring analysis of gene sets related to the oxidative stress response in the scRNA‐seq dataset. Wilcox test. *p* < .001 (***). (E) Expression of ROS metabolism‐related enzyme genes in different cell types based on the scRNA‐seq dataset. Red‐ and green‐marked cell types are, respectively, termed ‘heterogeneous cell types’ with increased abundance correlating to poorer and better patient prognosis. The red‐marked gene, GPX4, is a focal point in this study and is associated with ROS metabolism. (F) Expression analysis of GPX4 in the GSE39582 dataset, Wilcox test. *p* < .001 (***). (G) GPX4 expression analysis in the TCGA dataset, Wilcox test. *p* < .001 (***). (H and I) Fluorescence intensity results of GPX4 in 52 pairs of CRC and normal tissues, Wilcox test. *p* < .001 (***). (J and K) Comparison of GPX4 expression in plasma cells (CD138^+^), CD8^+^ T cells (CD8A^+^) and myeloids (CD11B^+^) in the CRC microenvironment. (L) Schematic representation of cells with different expression levels of ROS metabolism‐related enzymes (represented by GPX4) in the process of combating ROS in the CRC microenvironment.

We found that the ssGSEA scores of two oxidative stress‐related gene sets from the Molecular Signatures Database (MSigDB) in the tumour core were significantly higher than those in the border and normal tissues, indicating that the tumour core might have higher levels of oxidative stress than the border and normal tissues (Figure 2A,B). Furthermore, the scores of these gene sets in stromal cells, myeloids and endothelial cells were higher than those in most immune cells (Figure 2C,D). This suggests that these cells might have a stronger ROS metabolic capacity than immune cells. Therefore, we inferred that the expression of oxidative stress‐related genes (OSRGs) in these heterogeneous cell types might have been altered and involved in tumour recurrence. Then, we analysed the expression of the SOD, PRX and GPX families within these cell types and found that the expression levels of ROS‐metabolising enzymes were higher in stromal cells, myeloids and endothelial cells than in immune cells, particularly in plasma cells and CD8^+^ T cells (Figure 2E). Among these ROS metabolism enzymes, low GPX4 expression drives ferroptosis in CD8^+^ T cells in the TME, leading to antitumour immune suppression.^5^ Hence, we focused on GPX4 expression, which was high in CRC (Figure 2F–I). Myeloid cells, known for inhibiting other immune cells,^6^ exhibited significantly higher GPX4 expression than CD8^+^ T cells and plasma cells (Figure 2J,K). The abundance of GPX4‐positive cells could impact patient recurrence (Figure S2K–M). Therefore, besides the previously reported CD8^+^ T cells, low GPX4 expression in plasma cells and high GPX4 expression in myeloid cells may also be linked to TME heterogeneity and antitumour immune suppression (Figure 2L).

We identified 282 OSRGs from these ‘heterogeneous cell types’ (Figure 3A), and an additional 60 OSRGs differentially expressed between normal and tumour tissues (Figure 3B). The enrichment analysis results also suggested that the 60 identified OSRGs were involved in the oxidative stress response and tumorigenesis (Figure 3C).

**FIGURE 3:**
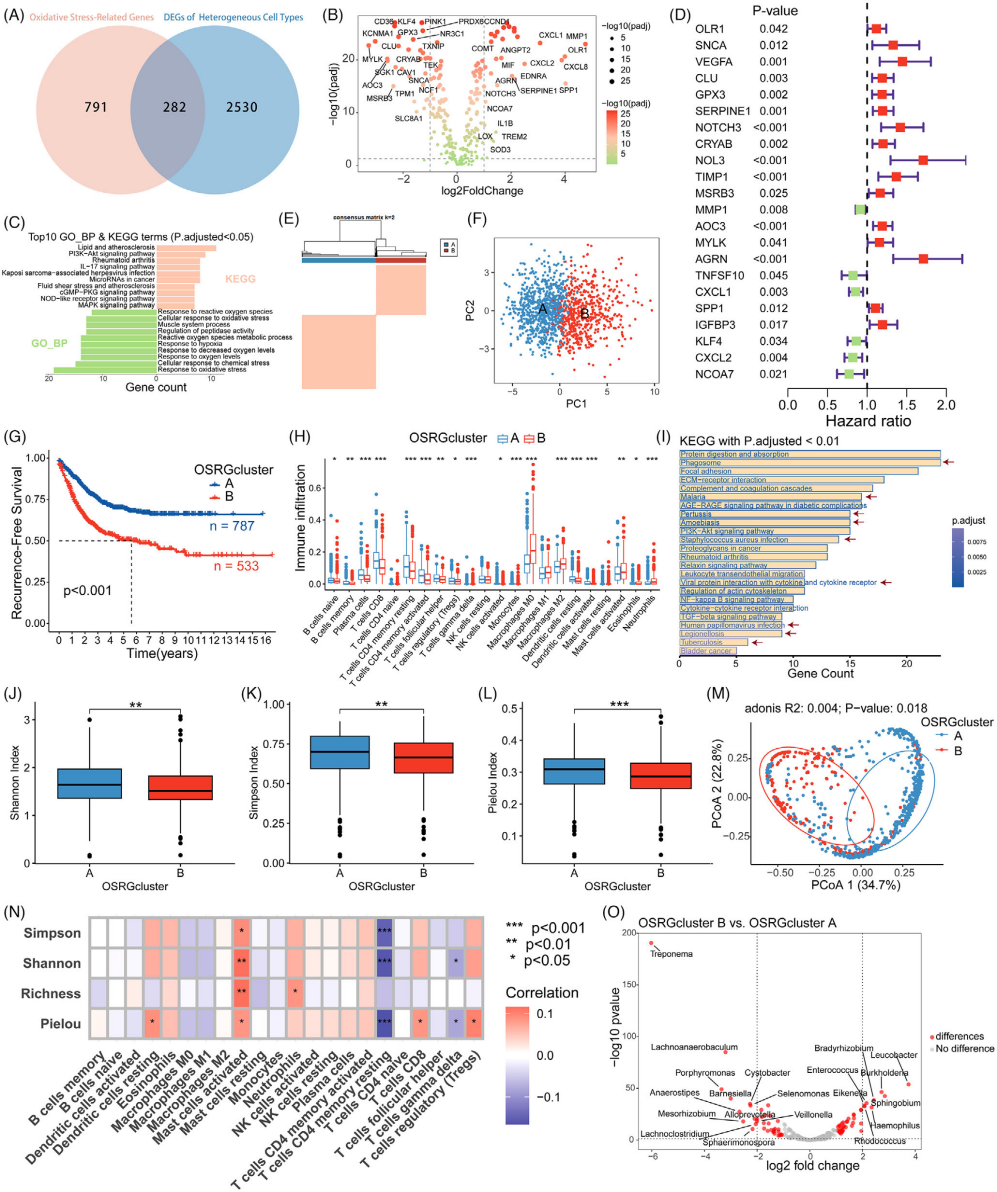
Molecular subtyping of CRC patients based on OSRG expression. (A) Number of differentially expressed OSRGs in ‘heterogeneous cell types’ in the scRNA‐seq dataset. (B) Analysis of differential gene expression between colorectal cancer and normal tissues based on the TCGA dataset. (C) GO and KEGG enrichment analysis of OSRGs. The top 10 terms are presented, organised by the count of enriched genes, following correction for *p* < .05. (D) Univariate Cox regression prognostic analysis of OSRGs based on the TCGA dataset. (E) Molecular subtype clustering of 1320 colorectal cancer patients, which was integrated from TCGA_CRC, GSE17536 and GSE39582 datasets, based on OSRGs related to prognosis. (F) PCA of OSRG expression in 1320 colorectal cancer patients based on prognosis. (G) Prognostic analysis of RFS among different OSRG clusters of 1320 colorectal cancer patients. (H) Differential analysis of immune cell infiltration levels among different OSRG clusters of 1320 colorectal cancer patients, in which FDR < .05 (*), FDR < .01 (**) and FDR < .001 (***). (I) KEGG pathways with *p* adjusted <.01 for differentially expressed genes between two distinct OSRG clusters. The arrow points to the pathway related to infectious diseases caused by pathogens. (J–L) Comparison of the α diversity of the intratumoural microbiome between the two different OSRG clusters based on the TCGA dataset. Wilcox test. *p* < .05 (*), *p* < .01 (**), *p* < .001 (***). (M) Comparison of the β diversity of the intratumoural microbiome between the two different OSRG clusters based on the TCGA dataset. (N) Analysis of the *Spearman* correlation between *α* diversity and immune cell infiltration levels based on the TCGA dataset. *p* < .05 (*), *p* < .01 (**), *p* < .001 (***). (O) Selection of differentially enriched microbial genera between OSRG cluster B and OSRG cluster A based on the TCGA dataset.

Moreover, using the TCGA_CRC dataset, we identified 22 recurrence‐related OSRGs (*p* < .05; Figure 3D) and categorised 1320 patients into OSRG clusters A and B based on their expression (Figure 3E,F). We observed significant differences in immune cell infiltration levels and recurrence among patients of distinct subtypes (Figure 3G,H).

Interestingly, differential gene enrichment analysis between the two subtypes revealed numerous infectious disease‐related pathways (Figure 3I), motivating further intratumoural microbiome analysis of these subtypes. OSRG cluster B exhibited significantly lower microbiome diversity than OSRG cluster A, and lower microbiome diversity was associated with patient recurrence.^7^ The microbial composition within the two subtypes differed significantly and was closely related to the levels of immune cell infiltration (Figure 3J–O).

To further uncover the clinical significance of the 22 recurrence‐related OSRGs in CRC, we randomly divided the TCGA_CRC cohort into a training cohort (*n* = 496) and a testing cohort (*n* = 122) using a 4:1 ratio. A 6‐OSRG signature was constructed using Lasso regression (Figure S3A–D):

Risk score = .101 × VEGFA + .076 × NOL3 + .010 × TIMP1 + .029 × AOC3 + .004 × SPP1 − .042 × CXCL2.

We demonstrated robust prediction performance in both internal validation (TCGA‐testing; Figure S3E) and external independent datasets (GSE39582 and GSE17536; Figure S3F,G). The performance remains robust even after integrating the entire dataset (Figure S3H). Figure S3I demonstrates the correlation between the expression of the 6‐OSRG signature and risk, while the high‐ and low‐risk statuses of all patients are depicted in Figure S3J,K. Moreover, we discovered a significant correlation between recurrence risk and the content of ‘heterogeneous cell types’ (Figure S3L,M).

The 6‐OSRG signature was further confirmed as an independent factor influencing patient prognosis through Cox regression analyses (Figure S4A,B). A comparison of various clinical features revealed significant associations between recurrence risk and tumour staging, molecular subtypes, hotspot gene mutations and PD‐L1 expression (Figure S4C–P). Stratified analysis of clinical features also suggested that the risk score may serve as an independent prognostic factor (Figure S5). Additionally, our analysis of overall survival (OS) indicated a decrease in OS among patients with high recurrence risk (Figure S6). Interestingly, the risk score also showed ideal discriminative performance for the OSRG clusters (Figure S7A–D). Consistent with prior research findings, patients carrying KRAS and TP53 mutations exhibited a poorer prognosis (Figure S7E,F).^8^ More importantly, the high‐risk group exhibited significantly lower diversity of the intratumour microbiome than the low‐risk group (Figure S7G–I). These results indicated that potential heterogeneity between high‐ and low‐risk patients may lead to varying outcomes under the same treatment regimen.

The expression patterns of the six OSRGs across various cell types in the single‐cell sequencing dataset are depicted in Figure S8A,B. Additionally, these scores exhibited a significant increase from normal tissue to the tumour core (*p* < .001; Figure S8C). The expression profiles of these six OSRGs in TCGA_CRC, GSE39582, and our mIHC cohort are shown in Figure S8D–G. The mIHC experiments validated the clinical significance of these six OSRGs in CRC recurrence (Figure S8H–M).

We also validated the performance of the 6‐OSRG signature in our mIHC cohort (Figure 4A–E). Moreover, we found that the protein expression of GPX4 was significantly higher in the high‐risk group than in the low‐risk group (*p* = .045; Figure 4F), indicating that GPX4 might serve as a synergistic gene contributing to an increased recurrence risk.^9^ The relationship between recurrence risk and the infiltration level of plasma cells, CD8^+^ T cells and myeloids was validated in the mIHC experiments (Figure 4G,H).

**FIGURE 4:**
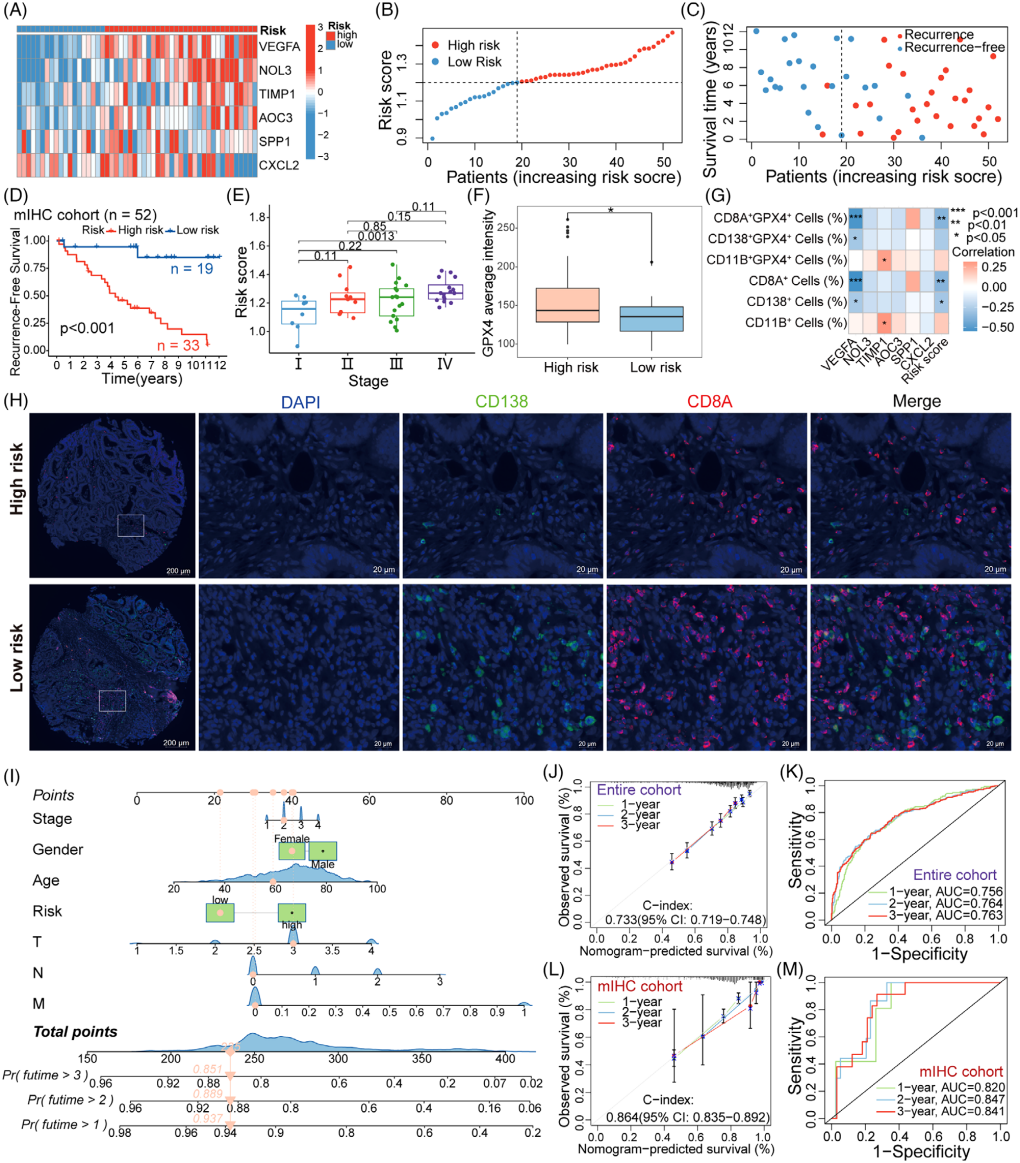
mIHC validation of the 6‐OSRG signature and construction of a nomogram. (A) Heatmap of 6‐OSRG expression in high‐ and low‐risk groups of the mIHC cohort. (B) Distribution of high‐ and low‐risk patients in the mIHC cohort. (C) Recurrence status of high‐ and low‐risk patients in the mIHC cohort. (D) Kaplan‒Meier survival analysis of high‐ and low‐risk patients in the mIHC cohort. (E) Association between stage and risk score of patients in the mIHC cohort. (F) GPX4 expression in high‐ and low‐risk patients of the mIHC cohort, *p* < .05 (*). (G) Correlation analysis between immune cell infiltration level and risk score in the mIHC cohort. (H) Representative images of plasma cells (CD138^+^) and CD8^+^ T cells (CD8A^+^) in high‐ and low‐risk patients of the mIHC cohort. (I) Nomogram developed for predicting patient RFS based on data from public datasets comprising 1320 patients. (J) Calibration curve for predicting 1‐, 2‐ and 3‐year RFS in the entire cohort using the nomogram. (K) ROC curve for predicting 1‐, 2‐ and 3‐year RFS in the entire cohort using the nomogram. (L) Calibration curve for predicting 1‐, 2‐ and 3‐year RFS in the mIHC cohort using the nomogram. (M) ROC curve for predicting 1‐, 2‐ and 3‐year RFS in the mIHC cohort using the nomogram.

To translate our research into practice, we developed a nomogram for CRC recurrence using data from 1320 patients (Figure 4I‐K).^10^ We validated its efficacy using our mIHC cohort and obtained consistent results (Figure 4L‐M). Recognising the limitations of traditional nomograms, we created the online assessment system ‘ColoPred’ (https://gaoxin.shinyapps.io/ColoPred/) based on OSRGs to facilitate the translation of prediction model into practical applications (Figure S9).

In conclusion, our study comprehensively elucidated the role of oxidative stress and related genes in the recurrence of CRC and their potential clinical utility.

## AUTHOR CONTRIBUTIONS

Jian‐Ping Cai designed and supervised the study. Xin Gao, Jin Li and Qing‐Yu Wang collected the data and performed the analyses. Zi‐Hui Wang, Si‐Jia Li, Lv‐Tao Zeng and Hong‐Lei Liu verified the underlying data. Xin Gao interpreted the results and wrote the manuscript. All authors had access to the study data, made the final decision on where to publish and contributed to the preparation, review and approval of the manuscript.

## CONFLICT OF INTEREST STATEMENT

The authors declare that they have no conflicts of interest.

## FUNDING INFORMATION

National Key R&D Program of China, Grant Number: 2018YFC2000300; Chinese Academy of Medical Sciences (CAMS) Innovation Fund for Medical Sciences, Grant Number: 2021‐1‐I2M‐050; National Natural Science Foundation of China, Grant Number: 82170856

## ETHICS STATEMENT

The colorectal cancer samples were collected during routine surgical procedures with written informed consent, in accordance with the approval of the local medical institution (Shanghai Tongren Hospital Ethics Committee) (Tongren Ethics Review 2020‐035‐01).

## Supporting information

Supporting Information

Supporting Information

Supporting Information

## Data Availability

The data that support the findings of this study are openly available in ‘figshare’ at https://doi.org/10.6084/m9.figshare.24465799
