# Supplementary material for: Exploring the potential role of oxidative stress‐related genes in colorectal cancer recurrence and establishing a recurrence assessment system based on single‐cell and bulk RNA‐seq analysis
Source: Clin Transl Med. 2024 Feb 8;14(2):e1577. doi: 10.1002/ctm2.1577 (PMC10851094; doi:10.1002/ctm2.1577)
Supplement: Supplementary file 1 — Supporting Information [file CTM2-14-e1577-s002.docx]

**Exploring the Potential Role of Oxidative Stress-Related Genes in Colorectal Cancer Recurrence and Establishing a Recurrence Assessment System Based on Single-Cell and Bulk RNA-seq Analysis**

**Materials and methods**

***Acquisition and basic information of public datasets***

The CRC scRNA-seq dataset GSE144735 ^1^, along with the CRC bulk RNA-seq datasets GSE17536 ^2^ and GSE39582 ^3^, were obtained from the publicly accessible Gene Expression Omnibus (GEO) database (https://www.ncbi.nlm.nih.gov/geo/), which is a valuable resource for transcriptome data. The GSE144735 dataset included 6 CRC core samples, 6 CRC border samples, and 6 normal colorectal mucosa samples. The GSE17536 dataset contained 177 CRC samples, and the GSE39582 dataset included 566 CRC samples and 19 normal colorectal mucosa samples. We also obtained transcriptome data for colon adenocarcinoma/rectum adenocarcinoma (COAD/READ) from The Cancer Genome Atlas (TCGA) database (https://portal.gdc.cancer.gov/), which included 647 tumour tissues and 51 normal tissues. To avoid confusion, we referred to this dataset as "TCGA_CRC" in our study. The clinical information of the samples, including recurrence-free survival (RFS), gender, age, T stage, N stage, M stage, and pathological stage, was collected. Furthermore, KRAS, TP53, and MMR gene mutation data from CRC samples were obtained from both the GSE39582 dataset and TCGA database, while the MSI information of CRC patients was retrieved from The Cancer Immunome Database (TCIA) (https://tcia.at/) ^4^.

***Preparation and processing of gene sets***

To prepare the gene sets for our analysis, we obtained the oxidative stress-related genes (OSRGs) from the GeneCards database (https://www.genecards.org/) and The Molecular Signatures Database (MSigDB) (https://www.gsea-msigdb.org/gsea/msigdb/). To obtain a high-quality OSRG gene set, we selected the top 10% of genes with a relevance score > 6 in the GeneCards results ^5^, resulting in 1027 OSRGs. From the MSigDB database, we chose the "GOBP_RESPONSE_TO_OXIDATIVE_STRESS" gene set, which contained 436 OSRGs. These two gene sets were combined and deduplicated, resulting in a final gene set of 1073 OSRGs. The gene set scores in each dataset were calculated using the "ssGSEA" algorithm implemented in the GSVA package.

***Single-cell RNA-seq data analysis***

The CRC scRNA-seq dataset GSE144735 was generated using the GPL24676 Illumina NovaSeq 6000 platform. To ensure high-quality data, we applied rigorous filtering criteria using Seurat (version 4.1.0) to remove low-quality cells. Specifically, we required each gene to be expressed in at least 3 cells and each cell to express at least 250 genes. We then calculated the percentages of mitochondrial genes and rRNA using the "PercentageFeatureSet" function and removed cells that did not meet the quality control standards of mitochondrial gene content < 15%, RNA number > 500, and RNA count > 1000. After normalizing the expression data using the LogNormalize method in the "NormalizeData" function, we applied the "FindVariableFeatures" function to identify hypervariable genes. The data were normalized and centralized using the "ScaleData" function, followed by principal component analysis (PCA). The parameters for t-distributed stochastic neighbour embedding (t-SNE) analysis were set to Dim = 40 and resolution = 0.3. The identification of differentially expressed genes in each cluster as marker genes was performed using the "FindAllMarkers" function, with logFC > 0.3, a minimum expression ratio of differential genes (min.pct) of 0.25, and an adjusted *P* value of <0.05 as the criteria. We performed cell annotation by combining manual annotation with the singleR package (version 1.6.1). We constructed a 2x2 contingency table for each cell type in the tumour core and border and used Fisher's test (two-sided) to calculate the *P* value for the difference in cell number and the corresponding fold change (FC) between the core and border. *P* values were corrected using the false discovery rate (FDR) method. Differences in cell content between the core and border were considered significant if FDR < 0.05, with FC > 0 indicating an increase in core content and FC < 0 indicating a decrease. We utilized the CellChat package to conduct intercellular communication analysis, which provides a comprehensive framework for inferring cell‒cell communication networks and identifying putative communication pairs between different cell types. In our study, 'heterogeneous cell types' were defined as cell types with significantly different abundances between the core and border of tumours, and their abundance levels were significantly correlated with patient prognosis.

***Bulk RNA-seq analysis***

To integrate and harmonize a total of 1365 CRC samples from the TCGA_CRC, GSE17536, and GSE39582 datasets, the "Combat" algorithm was applied to remove batch effects ^6^. After excluding patients with incomplete RFS information, we obtained a bulk RNA-seq matrix consisting of 1320 CRC patients. Comprehensive clinical information can be found in **Table S1**. To assess the level of heterogeneous cell types in these patients, we utilized the "ssGSEA" algorithm to calculate the score of the top 10 cell marker genes identified from scRNA-seq data ^7^. Furthermore, the "CIBERSORT" algorithm was employed to evaluate the proportion of immune cell infiltration in each sample. We utilized the survival package to conduct a Kaplan‒Meier survival analysis. The limma package was utilized to identify differentially expressed OSRGs in TCGA_CRC, applying a filtering criterion of |logFC| > 1 and an adjusted *P* value < 0.05. Functional annotation was conducted using the clusterProfiler package for Gene Ontology (GO) and Kyoto Encyclopedia of Genes and Genomes (KEGG) pathway enrichment analysis with a significance level of adjusted *P* < 0.05.

***Identification of prognosis-related OSRGs and molecular subtyping of CRC patients***

We performed a comprehensive analysis of differentially expressed OSRGs in 1320 CRC patients. Prognosis-related OSRGs were identified through univariate Cox regression analysis using the survival package. The ConsensusClusterPlus package was then utilized to perform consensus clustering analysis based on the expression profiles of these identified prognosis-related OSRGs in the 1320 CRC patients, which enabled the identification of distinct OSRGclusters with differential expression patterns. Differential expression analysis of genes between different OSRGclusters was performed using the limma package. The criteria for differential gene selection were set as |logFC| > 1 and an adjusted *P* < 0.05.

***Colorectal cancer microbiome data collection and processing***

We obtained standardized microbial abundance profiles from the study by Poore et al. ^8^. Their research involved the utilization of raw data from TCGA samples, including whole-genome and transcriptome data, to identify microbial reads and quantify microbial abundances. Microbial α diversities were assessed using the vegan package in R. Bray‒Curtis distance was calculated, and principal coordinate analysis (PCoA) was visualized using the ggplot2 and ggalt packages. Differential analysis of microbial abundance between different OSRGclusters was performed using the DESeq2 package. The criteria for selecting differentially abundant microbes were set as |logFC| > 1 and an adjusted *P* < 0.05.

***Establishment and evaluation of an OSRG signature***

The "createDataPartition" function of the caret package was utilized to randomly partition TCGA_CRC patients into a training and a testing cohort at a ratio of 4:1. We utilized the glmnet package to conduct Lasso regression analysis on the training cohort. Subsequently, a tenfold cross-validation procedure was employed to accurately estimate the model's predictive performance and determine the optimal regularization parameter. This allowed us to establish an OSRG signature that can be used to predict RFS in CRC patients. The risk score for each patient was determined using the following formula:

risk score =∑coefficient (GeneN) × expression (GeneN).

The coefficients in the formula were obtained from Lasso regression. Patients were classified into high-risk and low-risk groups based on the median risk score obtained from the training cohort. This cut-off value was used to divide patients from all cohorts into two groups. The predictive power of the established OSRG signature was further validated in the testing cohort as well as in two independent cohorts (GSE17536 and GSE39582). Univariate and multivariate Cox regression analyses were employed to investigate whether the OSRG signature was independent of other clinical factors. The regplot package was utilized to draw a nomogram, and the concordance index (C-index) of the nomogram was calculated. Additionally, a web-based prognosis prediction tool was developed using the DynNom and shiny packages and deployed on the shinyapps platform (www.shinyapps.io/) ^9^.

***Multiplex immunohistochemistry (mIHC) experiment***

The AlphaTSA Multiplex IHC Kit (AlphaTSA 7, AXT37100031, Beijing, China) was utilized to perform the mIHC experiment in this study. Paraffin sections were baked in a constant temperature oven at 60°C for 120 minutes, followed by deparaffinization and hydration. The sections were then washed twice with distilled water, subjected to microwave antigen retrieval, cooled, and washed three times with TBST. Primary antibodies were added and incubated at 37°C for 1 hour. After three washes with TBST, the sections were incubated with the secondary antibodies provided in the kit at 37°C for 10 minutes. Fluorescence staining was then performed, and single cells were identified using DAPI nuclear staining. Finally, a mounting medium was applied to the sections. The images were acquired using the ZEISS Axioscan7 whole slide imaging system, and ZEN software (version 3.3) was used to analyse the cell positivity rate and measure the average fluorescence intensity.

We designed two antibody panels (**Table S2**) and used two tissue microarray slides from the same patients (purchased from Shanghai Liaoding Biotechnology Co., Ltd.). Three extra colon tissues that included simultaneous normal, tumour border, and tumour core tissues were included in panel 1. We designated the 52 CRC samples for mIHC experiments as the 'mIHC cohorts.' Additionally, to ensure alignment with GSE144735 in defining the border of our tumour slices ^1^, with guidance from a pathologist, we defined tumour tissue within 1 mm of the normal tissue as the 'tumour border' and tumour tissue beyond 1 mm from the normal tissue as the 'tumour core.'

***Statistical analysis***

Prognosis analysis was performed using the Kaplan‒Meier method, and the log-rank test was used to determine if differences were significant. The correlations between the two variables were examined by Spearman correlation analysis. Wilcoxon test were employed for differential analysis between two groups. Paired sample comparisons were performed using paired t-tests. Statistical analysis was performed using R software (version 4.1.2). A *P* value of less than 0.05 was considered statistically significant for the above results.

**REFERENCES**

1. Lee HO, Hong Y, Etlioglu HE, et al. Lineage-dependent gene expression programs influence the immune landscape of colorectal cancer. *Nat Genet*. 2020;52:594-603. doi:10.1038/s41588-020-0636-z

2. Smith JJ, Deane NG, Wu F, et al. Experimentally derived metastasis gene expression profile predicts recurrence and death in patients with colon cancer. *Gastroenterology*. 2010;138:958-968. doi:10.1053/j.gastro.2009.11.005

3. Marisa L, de Reyniès A, Duval A, et al. Gene expression classification of colon cancer into molecular subtypes: characterization, validation, and prognostic value. *PLoS Med*. 2013;10:e1001453. doi:10.1371/journal.pmed.1001453

4. Charoentong P, Finotello F, Angelova M, et al. Pan-cancer Immunogenomic Analyses Reveal Genotype-Immunophenotype Relationships and Predictors of Response to Checkpoint Blockade. *Cell Rep*. 2017;18:248-262. doi:10.1016/j.celrep.2016.12.019

5. Qiu X, Hou QH, Shi QY, et al. Identification of Hub Prognosis-Associated Oxidative Stress Genes in Pancreatic Cancer Using Integrated Bioinformatics Analysis. *Front Genet*. 2020;11:595361. doi:10.3389/fgene.2020.595361

6. Song W, Ren J, Xiang R, et al. Identification of pyroptosis-related subtypes, the development of a prognosis model, and characterization of tumor microenvironment infiltration in colorectal cancer. *Oncoimmunology*. 2021;10:1987636. doi:10.1080/2162402X.2021.1987636

7. Wu Y, Yang S, Ma J, et al. Spatiotemporal Immune Landscape of Colorectal Cancer Liver Metastasis at Single-Cell Level. *Cancer Discov*. 2022;12:134-153. doi:10.1158/2159-8290.CD-21-0316

8. Poore GD, Kopylova E, Zhu Q, et al. Microbiome analyses of blood and tissues suggest cancer diagnostic approach. *Nature*. 2020;579:567-574. doi:10.1038/s41586-020-2095-1

9. Jalali A, Alvarez-Iglesias A, Roshan D, et al. Visualising statistical models using dynamic nomograms. *PLoS One*. 2019;14:e0225253. doi:10.1371/journal.pone.0225253
