# Supplementary material for: Exploring the potential role of oxidative stress‐related genes in colorectal cancer recurrence and establishing a recurrence assessment system based on single‐cell and bulk RNA‐seq analysis
Source: Clin Transl Med. 2024 Feb 8;14(2):e1577. doi: 10.1002/ctm2.1577 (PMC10851094; doi:10.1002/ctm2.1577)
Supplement: Supplementary file 2 — Supporting Information [file CTM2-14-e1577-s003.docx]

**Table S1 Clinical characteristics of patients with colorectal cancer**

|  | **TCGA** | **GSE17536** | **GSE39582** | **mIHC** |
| --- | --- | --- | --- | --- |
|  | **n=618** | **n=145** | **n=557** | **n=52** |
| **Gender**: |  |  |  |  |
| Female | 289 (46.8%) | 69 (47.6%) | 250 (44.9%) | 26 (50.0%) |
| Male | 329 (53.2%) | 76 (52.4%) | 307 (55.1%) | 26 (50.0%) |
| **Age:** |  |  |  |  |
| Old (≥60) | 442 (71.5%) | 101 (69.7%) | 408 (73.2%) | 28 (53.8%) |
| Young (＜60) | 176 (28.5%) | 44 (30.3%) | 149 (26.8%) | 24 (46.2%) |
| **Status:** |  |  |  |  |
| Recurrence | 196 (31.7%) | 36 (24.8%) | 177 (31.8%) | 28 (53.8%) |
| Recurrence-free | 422 (68.3%) | 109 (75.2%) | 380 (68.2%) | 24 (46.2%) |
| **Stage:** |  |  |  |  |
| I | 105 (17.0%) | 24 (16.6%) | 32 (5.75%) | 8 (15.4%) |
| II | 228 (36.9%) | 55 (37.9%) | 260 (46.7%) | 11 (21.2%) |
| III | 181 (29.3%) | 56 (38.6%) | 201 (36.1%) | 17 (32.7%) |
| IV | 90 (14.6%) | 10 (6.90%) | 60 (10.8%) | 16 (30.8%) |
| unknown | 14 (2.27%) | 0 (0.00%) | 4 (0.72%) | 0 (0.00%) |
| **T stage:** |  |  |  |  |
| 0 | 0 (0.00%) | 0 (0.00%) | 1 (0.18%) | 0 (0.00%) |
| 1 | 20 (3.24%) | 0 (0.00%) | 11 (1.97%) | 1 (1.92%) |
| 2 | 105 (17.0%) | 0 (0.00%) | 43 (7.72%) | 7 (13.5%) |
| 3 | 421 (68.1%) | 0 (0.00%) | 361 (64.8%) | 37 (71.2%) |
| 4 | 70 (11.3%) | 0 (0.00%) | 118 (21.2%) | 7 (13.5%) |
| unknown | 2 (0.32%) | 145 (100%) | 23 (4.13%) | 0 (0.00%) |
| **N stage:** |  |  |  |  |
| 0 | 350 (56.6%) | 0 (0.00%) | 297 (53.3%) | 30 (57.7%) |
| 1 | 150 (24.3%) | 0 (0.00%) | 131 (23.5%) | 22 (42.3%) |
| 2 | 115 (18.6%) | 0 (0.00%) | 97 (17.4%) | 0 (0.00%) |
| 3 | 0 (0.00%) | 0 (0.00%) | 6 (1.08%) | 0 (0.00%) |
| unknown | 3 (0.49%) | 145 (100%) | 26 (4.67%) | 0 (0.00%) |
| **M stage:** |  |  |  |  |
| 0 | 458 (74.1%) | 0 (0.00%) | 474 (85.1%) | 35 (67.3%) |
| 1 | 87 (14.1%) | 0 (0.00%) | 61 (11.0%) | 17 (32.7%) |
| unknown | 73 (11.8%) | 145 (100%) | 22 (3.95%) | 0 (0.00%) |
| **MMR status:** |  |  |  |  |
| dMMR | 0 (0.00%) | 0 (0.00%) | 72 (12.9%) | 0 (0.00%) |
| pMMR | 0 (0.00%) | 0 (0.00%) | 439 (78.8%) | 0 (0.00%) |
| unknown | 618 (100%) | 145 (100%) | 46 (8.26%) | 52 (100%) |
| **KRAS Mutation:** |  |  |  |  |
| Mutation | 223 (36.1%) | 0 (0.00%) | 214 (38.4%) | 0 (0.00%) |
| Wild | 302 (48.9%) | 0 (0.00%) | 322 (57.8%) | 0 (0.00%) |
| unknown | 93 (15.0%) | 145 (100%) | 21 (3.77%) | 52 (100%) |
| **TP53 Mutation:** |  |  |  |  |
| Mutation | 306 (49.5%) | 0 (0.00%) | 188 (33.8%) | 0 (0.00%) |
| Wild | 219 (35.4%) | 0 (0.00%) | 156 (28.0%) | 0 (0.00%) |
| unknown | 93 (15.0%) | 145 (100%) | 213 (38.2%) | 52 (100%) |

| **Panel** | **Primary antibodies** | **Dilution** | **Source** |
| --- | --- | --- | --- |
| 1 | anti-CD8A | 1:100 | ab209775, Abcam |
|  | anti-CD138 | 1:2000 | ab128936, Abcam |
|  | anti-CD11B | 1:1000 | ab52478, Abcam |
|  | anti-GPX4 | 1:200 | ab125066, Abcam |
| 2 | anti-SPP1 | 1:200 | 22952-1-ap, Thermo Scientific |
|  | anti-AOC3 | 1:800 | 14365-1-ap, Thermo Scientific |
|  | anti-TIMP1 | 1:2000 | ab211926, Abcam |
|  | anti-CXCL2 | 1:100 | bs-1162R, Beijing Biosynthesis Biotechnology Co., Ltd. |
|  | anti-NOL3 | 1:2000 | ab288295, Abcam |
|  | anti-VEGFA | 1:500 | 19003-1-ap, Thermo Scientific |

**Table S2 The information of the primary antibodies**
