# Supplementary material for: Exploring the potential role of oxidative stress‐related genes in colorectal cancer recurrence and establishing a recurrence assessment system based on single‐cell and bulk RNA‐seq analysis
Source: Clin Transl Med. 2024 Feb 8;14(2):e1577. doi: 10.1002/ctm2.1577 (PMC10851094; doi:10.1002/ctm2.1577)
Supplement: Supplementary file 3 — Supporting Information [file CTM2-14-e1577-s001.docx]

**Exploring the Potential Role of Oxidative Stress-Related Genes in Colorectal Cancer Recurrence and Establishing a Recurrence Assessment System Based on Single-Cell and Bulk RNA-seq Analysis**

**Supplementary Figures and legends**

| **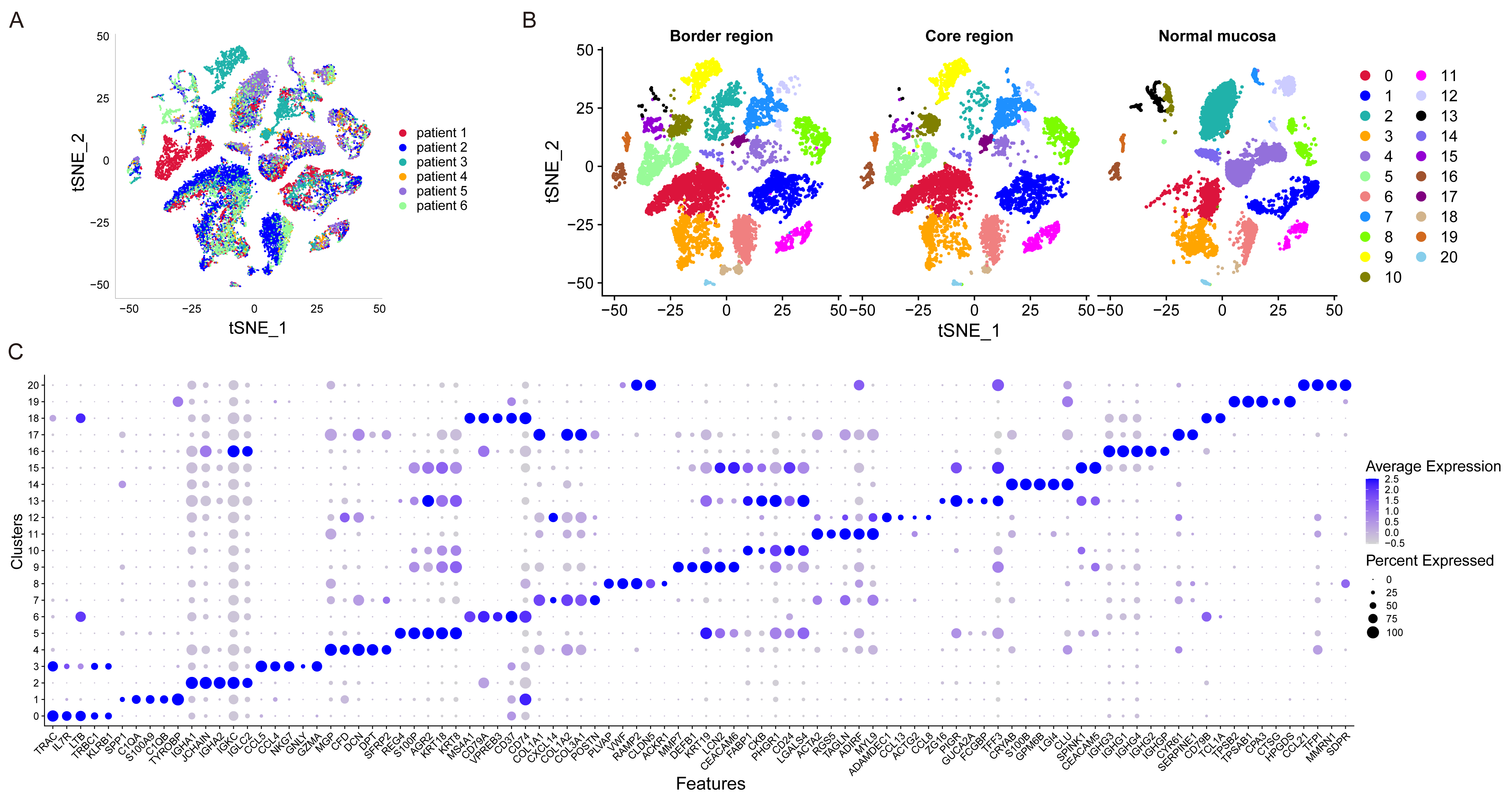** |
| --- |

**Figure S1. Clustering results of single-cell sequencing data and the relationship between GPX4-positive cells and recurrence in patients**

**A**, t-SNE clustering of single-cell sequencing data from six colorectal cancer patients. The cluster positions of the six samples largely overlap, indicating that batch effects between samples have been effectively corrected.

**B**, Cells were clustered using t-SNE and grouped according to their sample collection locations.

**C**, The top 5 marker genes expressed in each cluster. The point size indicates the proportion of cells expressing a specific gene within a particular cluster, while the color intensity indicates the average expression level of the gene in a particular cluster.

| **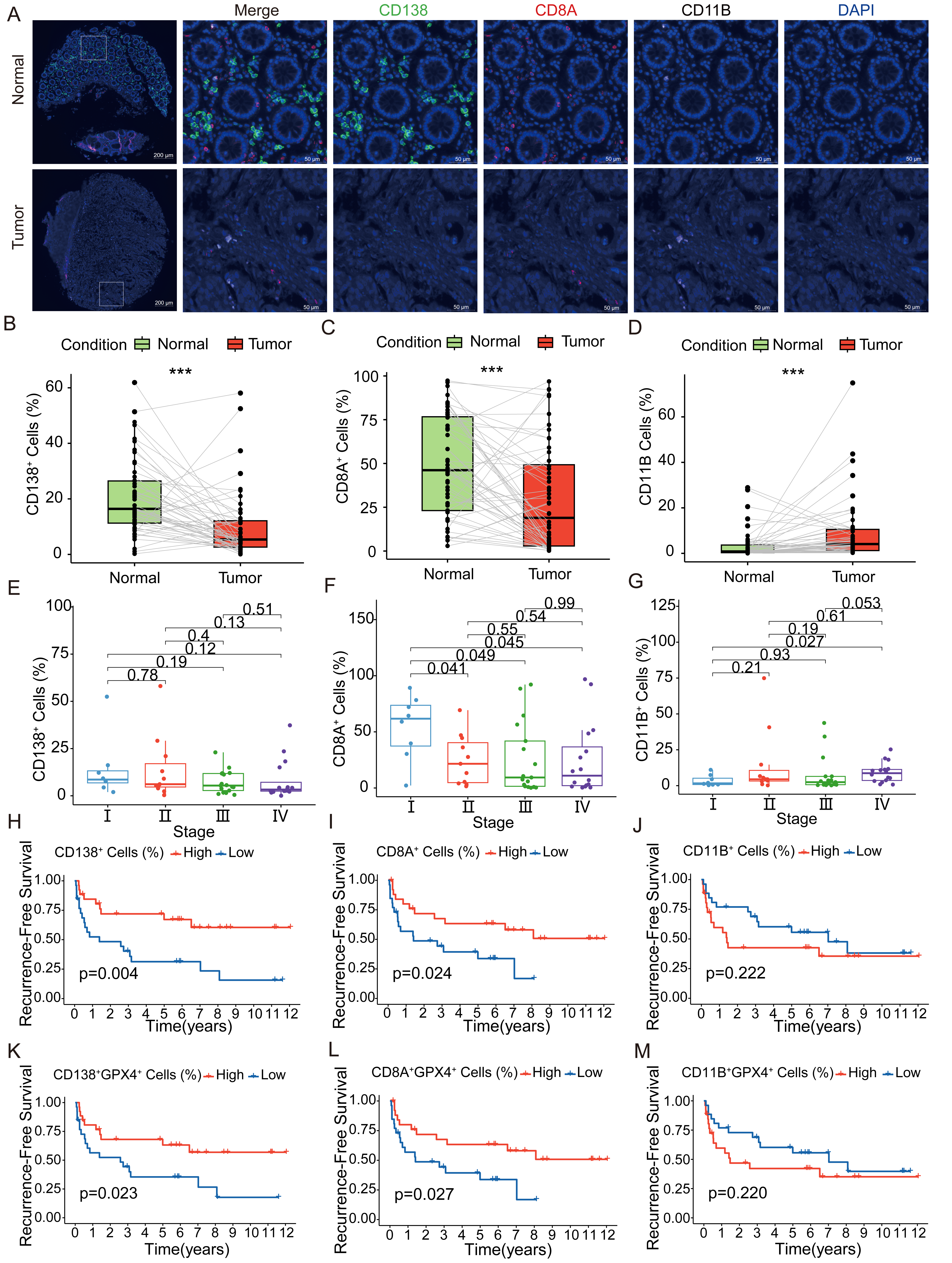** |
| --- |

**Figure S2. Confirmation of the impact of prognostic immune cell infiltration levels on patient recurrence through mIHC**

**A**, Representative images of normal and tumour tissue in tissue microarrays.

**B-D**, Analysis of differences in positive rates of the three cell markers between 52 pairs of normal and colorectal cancer tissues, in which the paired t-tests was used. *P* < 0.05 (*), *P* < 0.01 (**), and *P* < 0.001 (***).

**E-G**, Relationship between the positive rates of the three cell markers and pathological stage in 52 samples of colorectal cancer tissue.

**H-J**, Kaplan‒Meier curves of RFS analysis based on the positive rates of the three cell markers in 52 samples of colorectal cancer tissues.

**K‒M**, The ratio of immune cell types expressing GPX4 and its association with patient prognosis in terms of recurrence-free survival. After the localization of plasma cells, CD8^+^ T cells, and myeloids through CD138, CD8A, and CD11B, respectively, GPX4 staining was conducted. Signal collection analysis of coexpression cell positivity rates was performed using ZEN software (version 3.3).

.

.

| **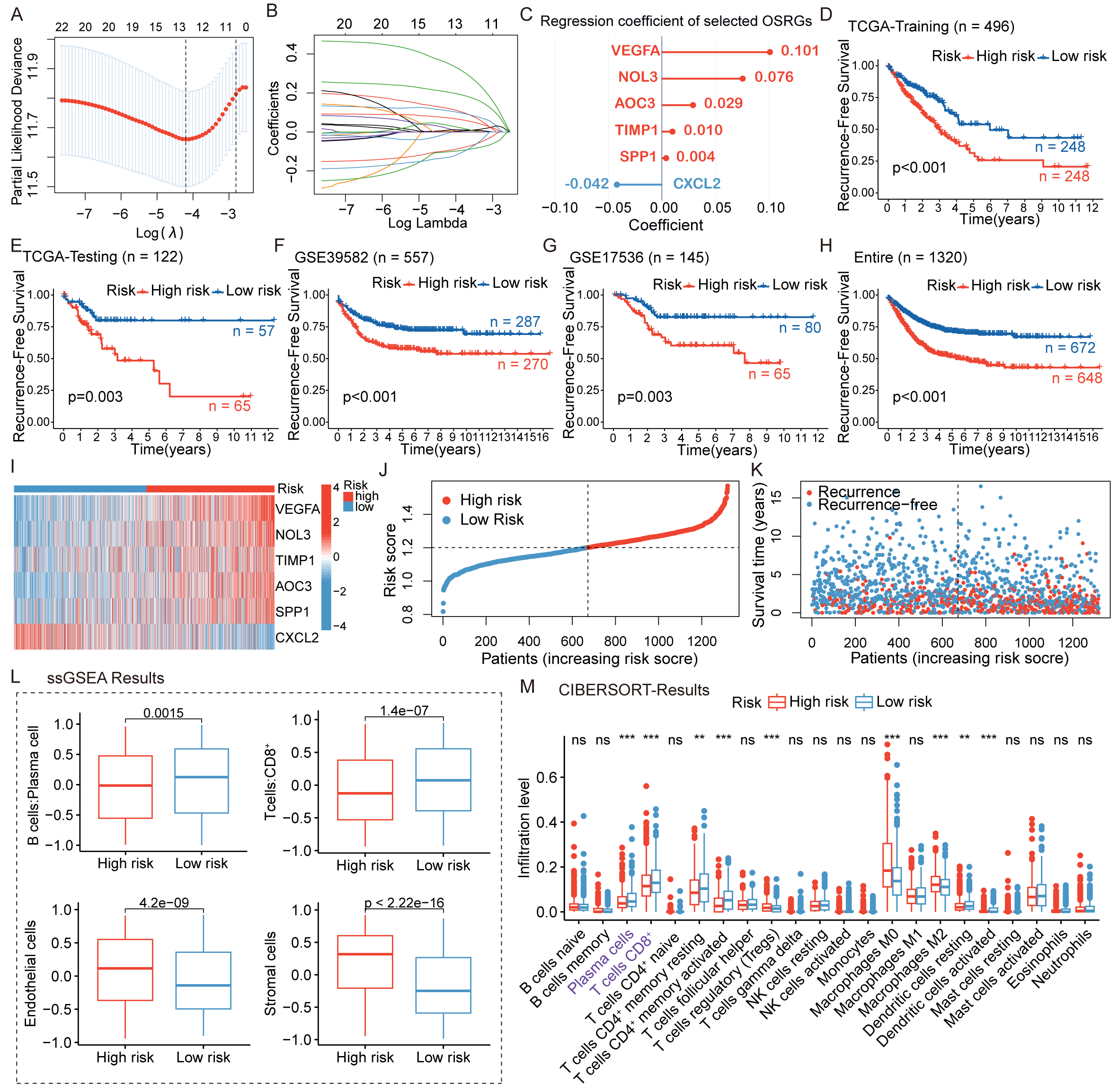** |
| --- |

**Figure S3. Development and validation of the 6-OSRG signature using public datasets**

**A**, Lasso regression cross-validation curve. The x-axis represents the penalty parameter log(lambda), and the y-axis represents the cross-validation error. The position of the left dashed line indicates the location of the minimum cross-validation error, while the position of the right dashed line represents the position of the simplest model (the trade-off between model simplicity and predictive performance).

**B**, Lasso regression coefficient plot. The x-axis represents log lambda values, and the y-axis represents the coefficients of the genes. As the lambda value increases, the gene coefficients tend to approach zero. Eventually, all coefficients become zero. When the coefficient of a gene becomes zero, the variation in that gene does not affect the risk values of the samples.

**C**, Lasso regression coefficients of the selected 6 OSRGs.

**D**. Prognostic analysis of high- and low-risk patients in the training cohort.

**E**, Prognostic analysis of high- and low-risk patients in the testing cohort.

**F**, Prognostic analysis of high- and low-risk patients in GSE39582.

**G**, Prognostic analysis of high- and low-risk patients in GSE17536.

**H**, Prognostic analysis of 1320 high- and low-risk patients integrated from all datasets (entire cohort).

**I**, Expression of the 6 OSRGs in high- and low-risk patients in the entire cohort.

**J-K**, Recurrence status of patients in the entire cohort with changes in risk score.

**L**, Correlation between cell content evaluated by ssGSEA and risk scores in the entire cohort (*P* < 0.05).

**M**, Correlation between cell content evaluated by the CIBERSORT algorithm and risk scores in the entire cohort. The immune cell types marked in blue are the specific cell types of primary interest in this study. FDR < 0.05 (*), FDR < 0.01 (**), and FDR < 0.001 (***).

| **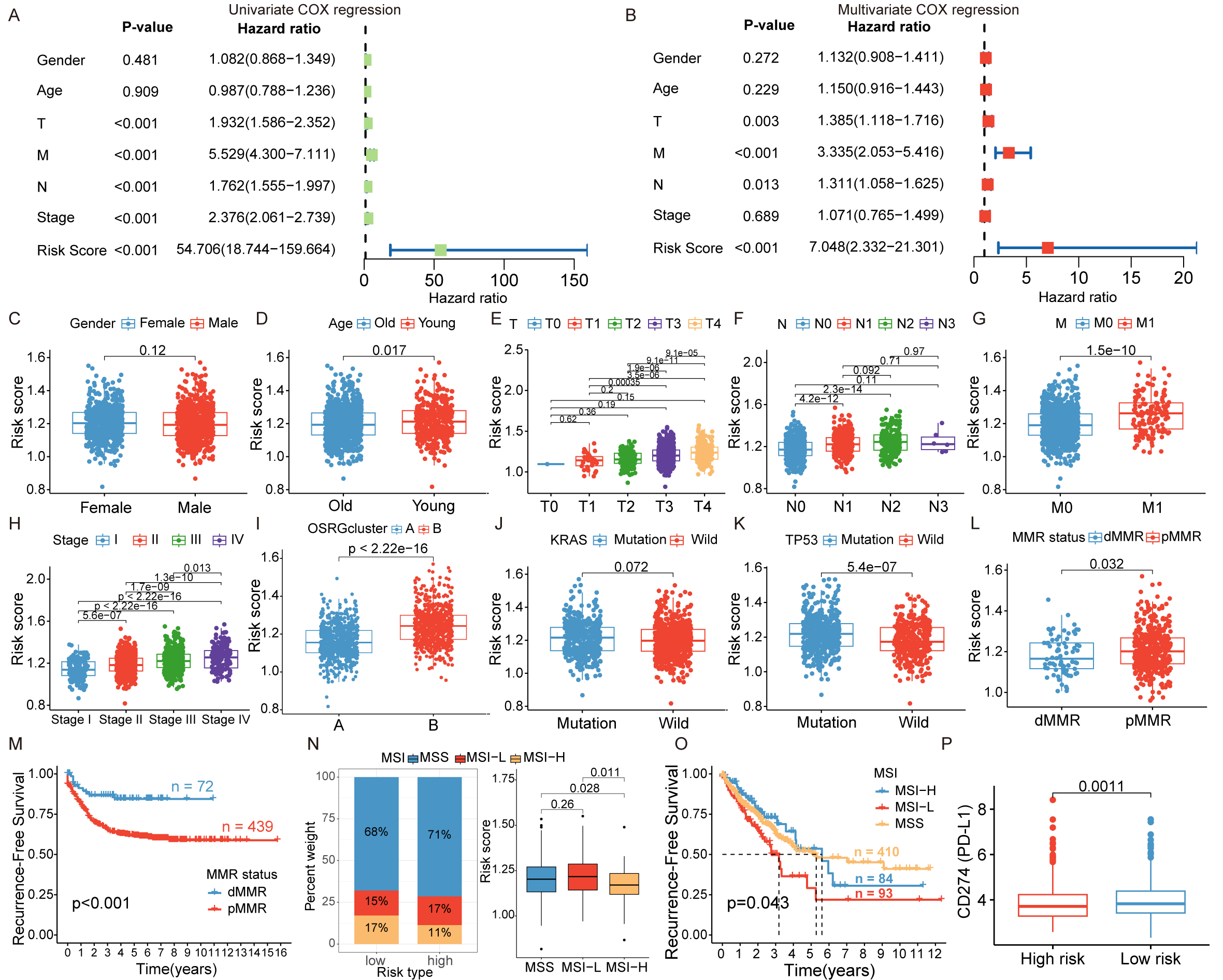** |
| --- |

**Figure S4. Correlation analysis of risk score and characteristics of patients with colorectal cancer**

**A-B**, Univariate and multivariate Cox regression analyses were performed to evaluate the significance of the risk score and other clinical factors in predicting prognosis in colorectal cancer patients of the entire cohort.

**C-K**, The risk scores were compared among subgroups of patients in the entire cohort categorized by gender, age, T stage, N stage, M stage, pathological stage, OSRGcluster, KRAS mutation status, and TP53 mutation status.

**L**, The risk scores were compared between mismatch repair deficient (dMMR) and mismatch repair proficient (pMMR) patients in the entire cohort.

**M**, Kaplan‒Meier survival analysis was performed to compare the prognoses of dMMR and pMMR patients in the entire cohort.

**N**, The risk scores were compared among patients in the entire cohort with different types of microsatellite instability (MSI).

**O**, Kaplan‒Meier survival analysis was conducted to compare the prognoses of patients in the entire cohort with different types of MSI.

**P**, The correlation between risk type and CD274 (PD-L1) expression in the entire cohort.

| 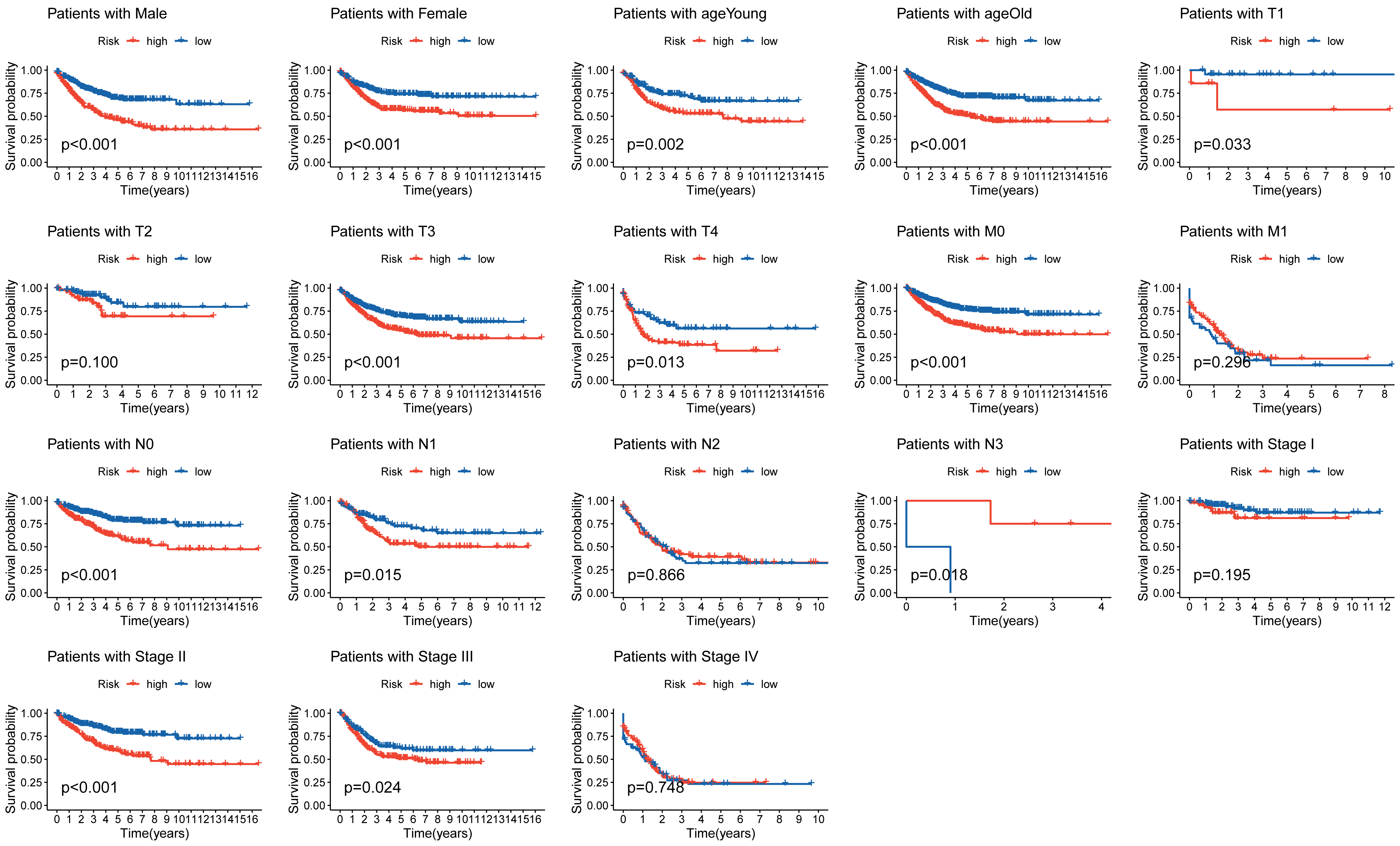 |
| --- |

**Figure S5. Recurrence-free survival stratification analysis of high- and low-risk patients in the entire cohort with different clinical characteristics**

These results confirm that risk scores can influence recurrence-free survival with the same clinical characteristics, suggesting that the assessment of prognosis by risk scores is independent of patients' clinical characteristics and may be an independent prognostic factor.

| **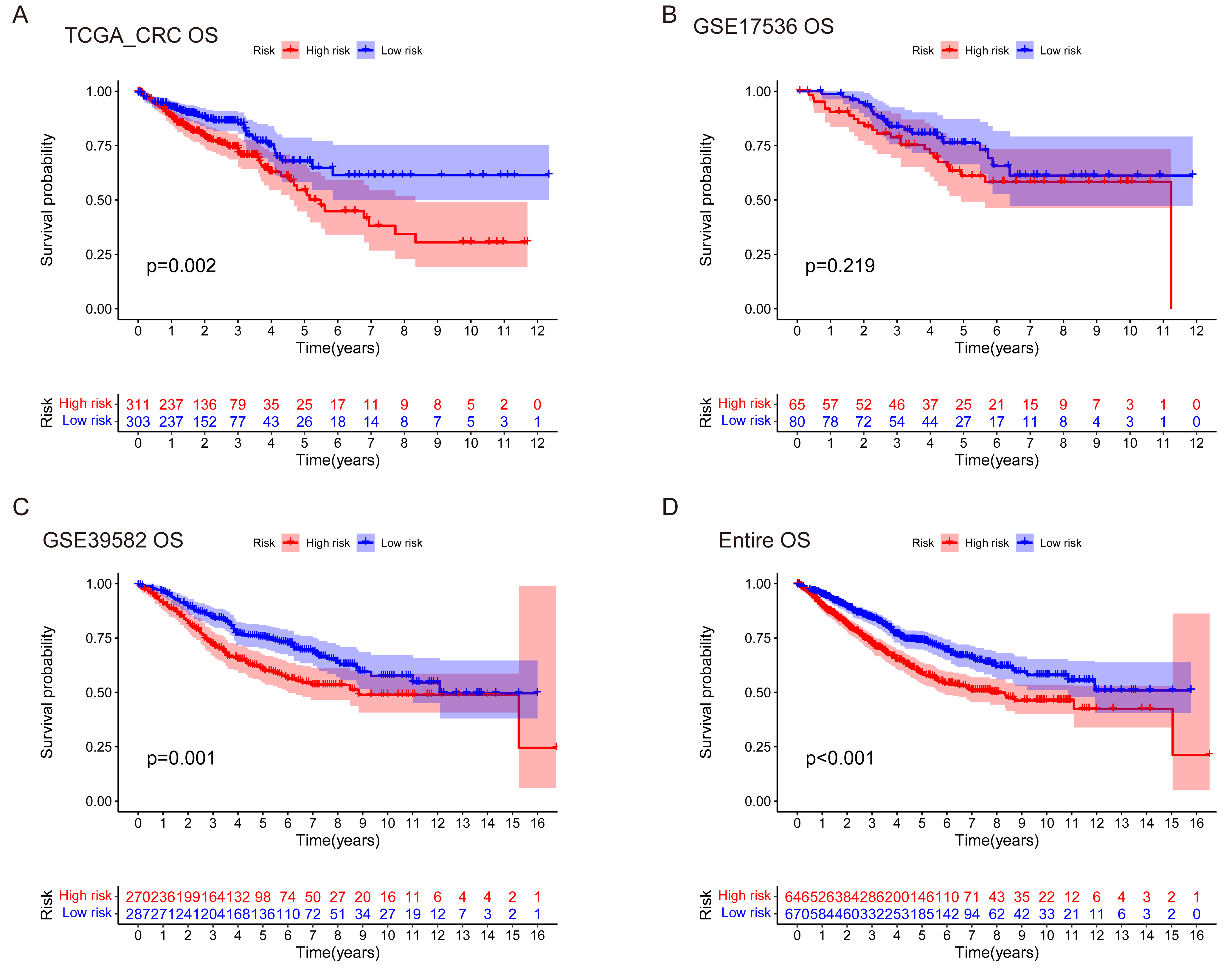** |
| --- |

**Figure S6. The predictive effect of the 6-OSRG signature on overall survival (OS)**

**A**, Overall survival prognosis analysis in the TCGA cohort by stratifying patients into high and low-risk groups using the 6-OSRG signature.

**B**, Overall survival prognosis analysis in the GSE17536 cohort by stratifying patients into high and low-risk groups using the 6-OSRG signature.

**C**, Overall survival prognosis analysis in the GSE39582 cohort by stratifying patients into high and low-risk groups using the 6-OSRG signature.

**D**, Overall survival prognosis analysis in the Entire cohort by stratifying patients into high and low-risk groups using the 6-OSRG signature.

| **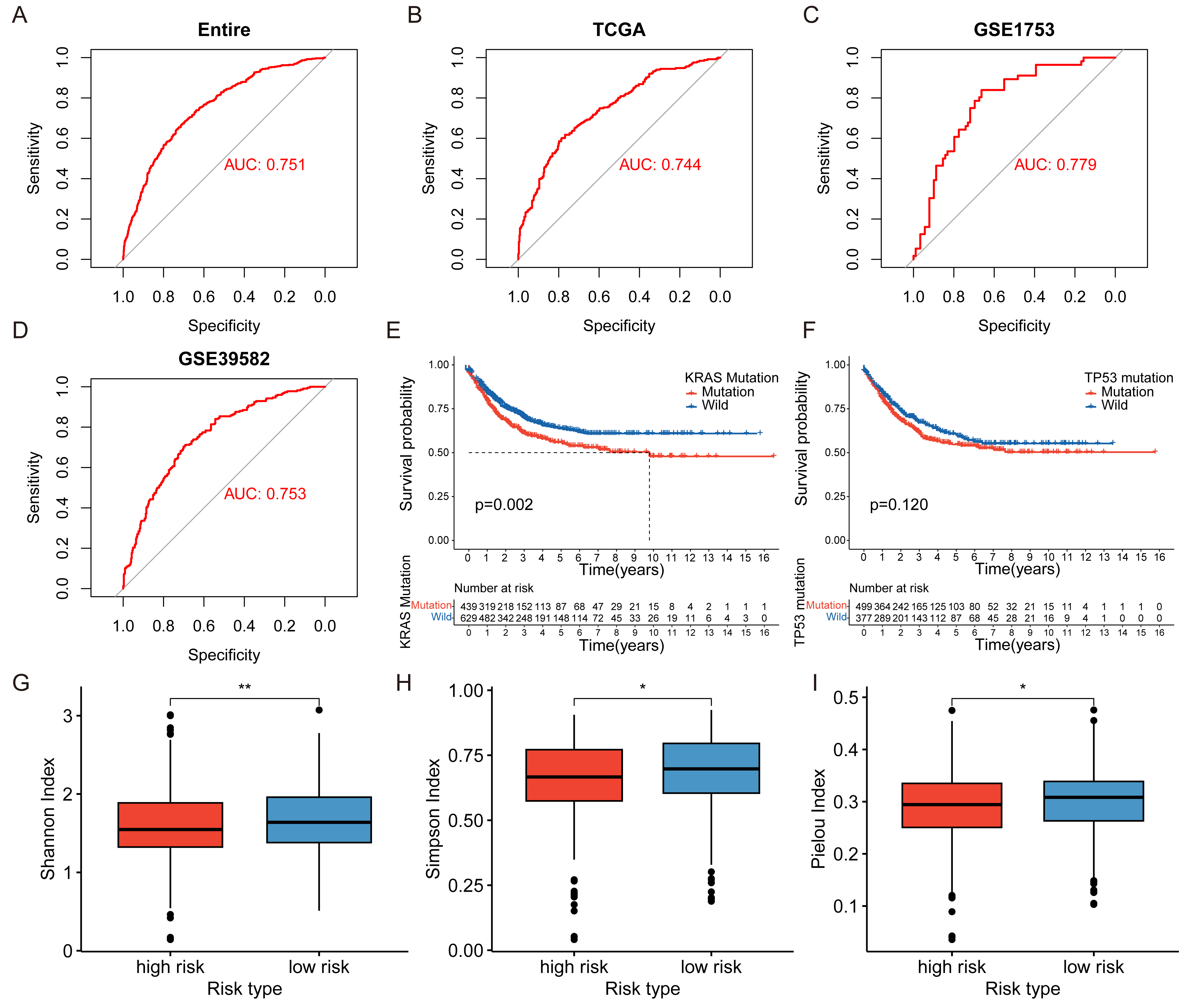** |
| --- |

**Figure S7. Evaluation of the diagnostic performance of the risk score for OSRGclusters and differential analysis of intratumoral microbiome composition among patients with different risk types.**

**A-D**, Evaluation of the diagnostic performance of the risk score for OSRG clusters.

**E-F**, Impact of KRAS and TP53 mutations on RFS in the entire cohort.

**G-I**, Differential analysis of intratumoral microbiome α diversity among patients in the TCGA cohort with different risk types. Wilcox test. *P* < 0.05(*), *P* < 0.01(**), *P* < 0.001(***).

| **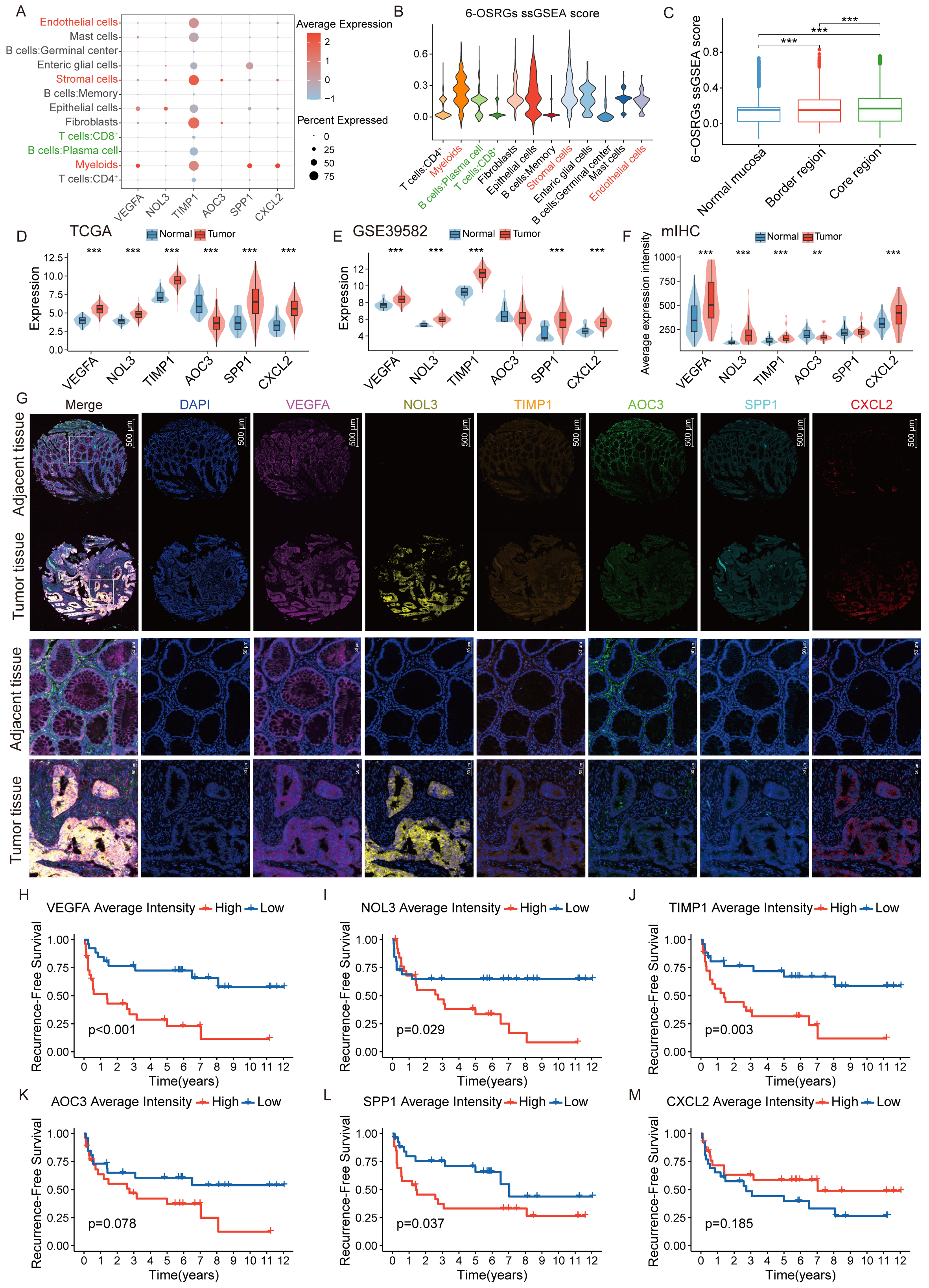** |
| --- |

**Figure S8. Expression analysis of 6 OSRGs**

**A**, Expression of 6 OSRGs in various cell types in the single-cell sequencing (scRNA-seq) dataset.

**B**, Calculation of the score for each cell type based on the expression of 6 OSRGs in the scRNA-seq dataset using the ssGSEA algorithm.

**C**, Comparison of ssGSEA algorithm scores for 6 OSRGs in different tissue types in the scRNA-seq dataset, Wilcox test. *P* < 0.001 (***).

**D-F**, Comparison of the expression of 6 OSRGs in normal and CRC tissues in TCGA cohort, GSE39582 cohort, mIHC cohort, Wilcox test. *P* < 0.05 (*), *P* < 0.01 (**), *P* < 0.001 (***).

**G**, Representative images of the expression of 6 OSRG in tumour and adjacent samples.

**H-M**, Kaplan‒Meier survival analysis of 6 OSRGs in the mIHC cohort.

| **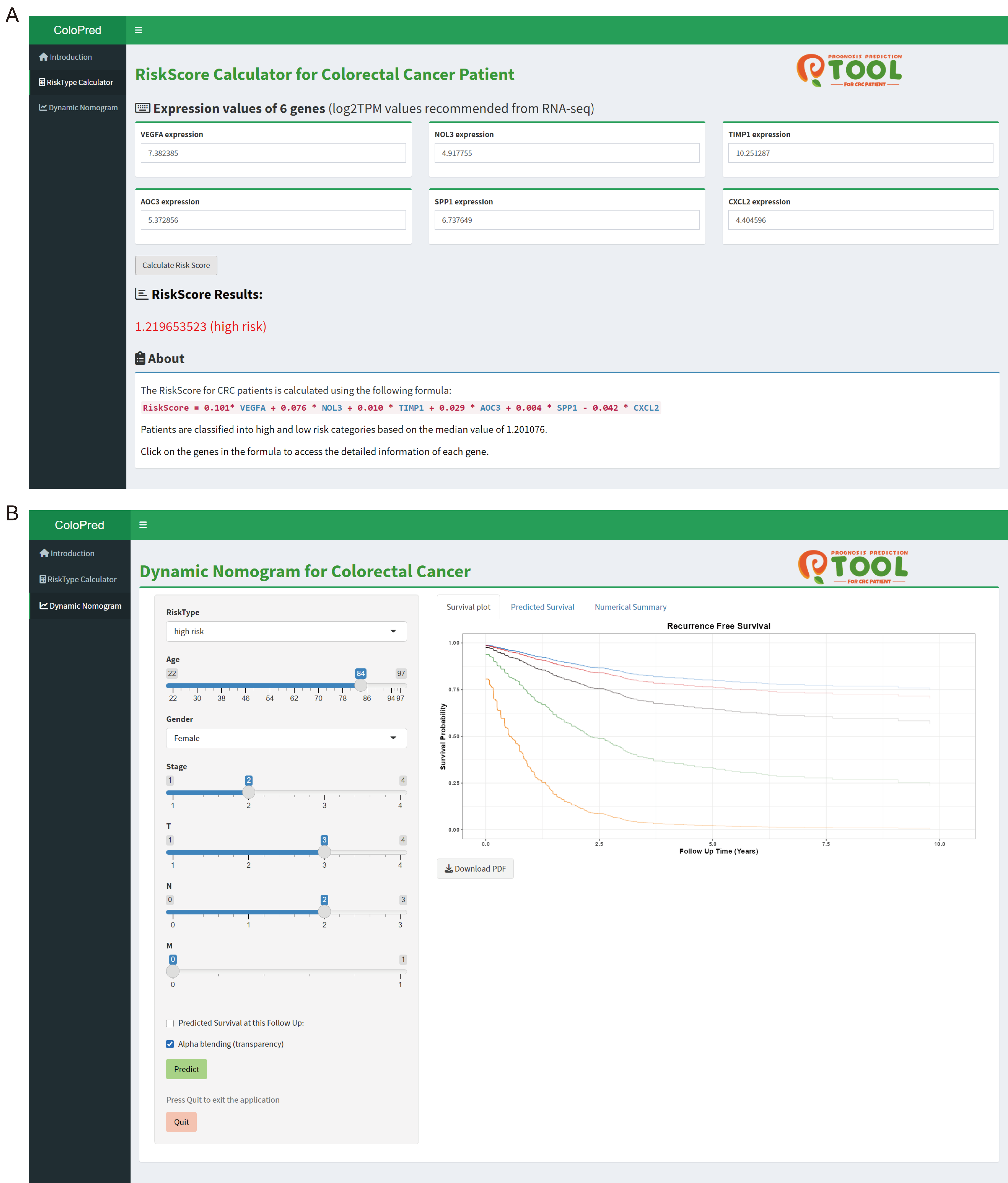** |
| --- |

**Figure S9. "ColoPred" operation interface diagram**

**A,** Calculator for colorectal cancer patient recurrence risk score and risk type.

**B,** Comprehensive colorectal cancer patient recurrence prediction tool incorporating multiple factors, including risk types.
